# A comparative study of short-term intensive and prolonged low-intensity simulation training on clinical competence among medical students

**DOI:** 10.3389/fmed.2026.1850843

**Published:** 2026-09-01

**Authors:** Yao Zhou, Nvwa Jin, Jinqiu Fu, Hanxiang Zhan

**Affiliations:** 1 Department of Pediatrics, Qilu Hospital of Shandong University, Jinan, Shandong, China; 2 Clinical Skills Center, Qilu Hospital of Shandong University (The First Clinical College), Jinan, Shandong, China; 3 Division of Pancreatic Surgery, Department of General Surgery, Qilu Hospital, Shandong University, Jinan, Shandong, China

**Keywords:** intensive simulation, medical education, medical students, skill mastery, skills competition

## Abstract

**Introduction:**

Clinical simulation training is widely used to improve procedural proficiency and patient safety; however, the relative effectiveness of different training schedules remains uncertain. This study compared clinical competence outcomes associated with short-term intensive and prolonged distributed simulation training among medical students.

**Methods:**

This prospective, non-randomized comparative cohort study included 48 fifth-year medical students. Based on their preferences, participants selected either a 15-day intensive simulation program (Group A, *n* = 24; approximately 8 h/day) or a 5-month distributed simulation program (Group B, *n* = 24; no more than 2 h/day). The total training duration was comparable between the groups. After training, both groups participated in team-based clinical skills competitions. Outcomes included scores from the Shandong Provincial College Student Medical Technology Skills Competition and structured post-training assessments of specialized knowledge, clinical skills, teamwork, emergency response capacity, and integrated professionalism completed by instructors and students.

**Results:**

Group A achieved higher competition scores than Group B in basic surgical procedures, internal medicine procedures, gynecological and pediatric procedures, emergency medical skills, and physician-patient communication skills (all *p* < 0.05). The between-group difference in comprehensive case-analysis skills was not statistically significant (*p* = 0.065). Group A also received higher instructor ratings and reported higher self-assessment scores across all evaluated domains than Group B (all *p* < 0.05).

**Discussion:**

Participation in short-term intensive simulation training was associated with better immediate competition performance and more favorable instructor and student ratings than participation in prolonged distributed training. However, because group allocation was preference-based and potentially important baseline variables were unavailable, the findings do not establish causal superiority or long-term benefits of intensive training. Concentrated, feedback-rich simulation may be useful when immediate technical and team performance is prioritized, but randomized studies incorporating retention and clinical-transfer outcomes are needed.

## 1 Introduction

In the ever-evolving field of medical education, the demand for highly competent and proficient medical professionals has reached unprecedented levels. As healthcare systems become increasingly complex, medical education must adapt to ensure that students acquire not only theoretical knowledge but also practical skills and professional attitudes (1, 2). Short-term intensive simulation training has emerged as a vital educational tool, providing a safe and controlled environment for medical undergraduates to practice and enhance their skills (3, 4).

Simulation-based education allows learners to engage with realistic clinical scenarios, integrate knowledge across disciplines, practice technical skills without risk to patients, and develop teamwork and communication (5, 6). However, the available evidence does not support a simple conclusion that greater training intensity is inherently better. Many studies have examined narrow or proximal outcomes, such as procedural confidence (7), technical proficiency (8), or performance in a specific simulated task, while fewer have compared alternative schedules using multidimensional outcomes. Moreover, immediate post-training or competition performance may reflect rehearsal, contextual similarity, and test familiarity rather than durable learning or transfer to patient care. Thus, the relevant gap is not merely whether simulation works, but how the spacing and intensity of practice are associated with different dimensions and time horizons of learning.

Our design is grounded in three established educational theories. Deliberate practice emphasizes repetitive, goal-oriented practice with immediate feedback (9, 10). Experiential Learning conceptualizes learning as a cycle of concrete experience, reflective observation, abstract conceptualization, and active experimentation; a distributed schedule may provide more time for reflection and application between sessions (11). Mastery Learning requires learners to meet explicit performance standards through assessment, feedback, and additional practice rather than progressing according to a fixed amount of time (12). These theories therefore suggest competing, testable expectations: concentrated training may support rapid gains in rehearsed performance, whereas spacing may support reflection and retention, and mastery depends on feedback and attainment standards rather than schedule alone. Guided by this framework, we compared immediate competition performance and multidimensional post-training ratings between students who selected intensive and distributed schedules. We hypothesized that the intensive group would show higher immediate performance, while recognizing that the design could not determine causal superiority or long-term retention (13). Through this investigation, it seeks to provide partial evidence that can optimize medical education practices, thereby assisting educational institutions and policymakers in making informed decisions.

## 2 Methods

### 2.1 Study design and participants

**Figure 1:**
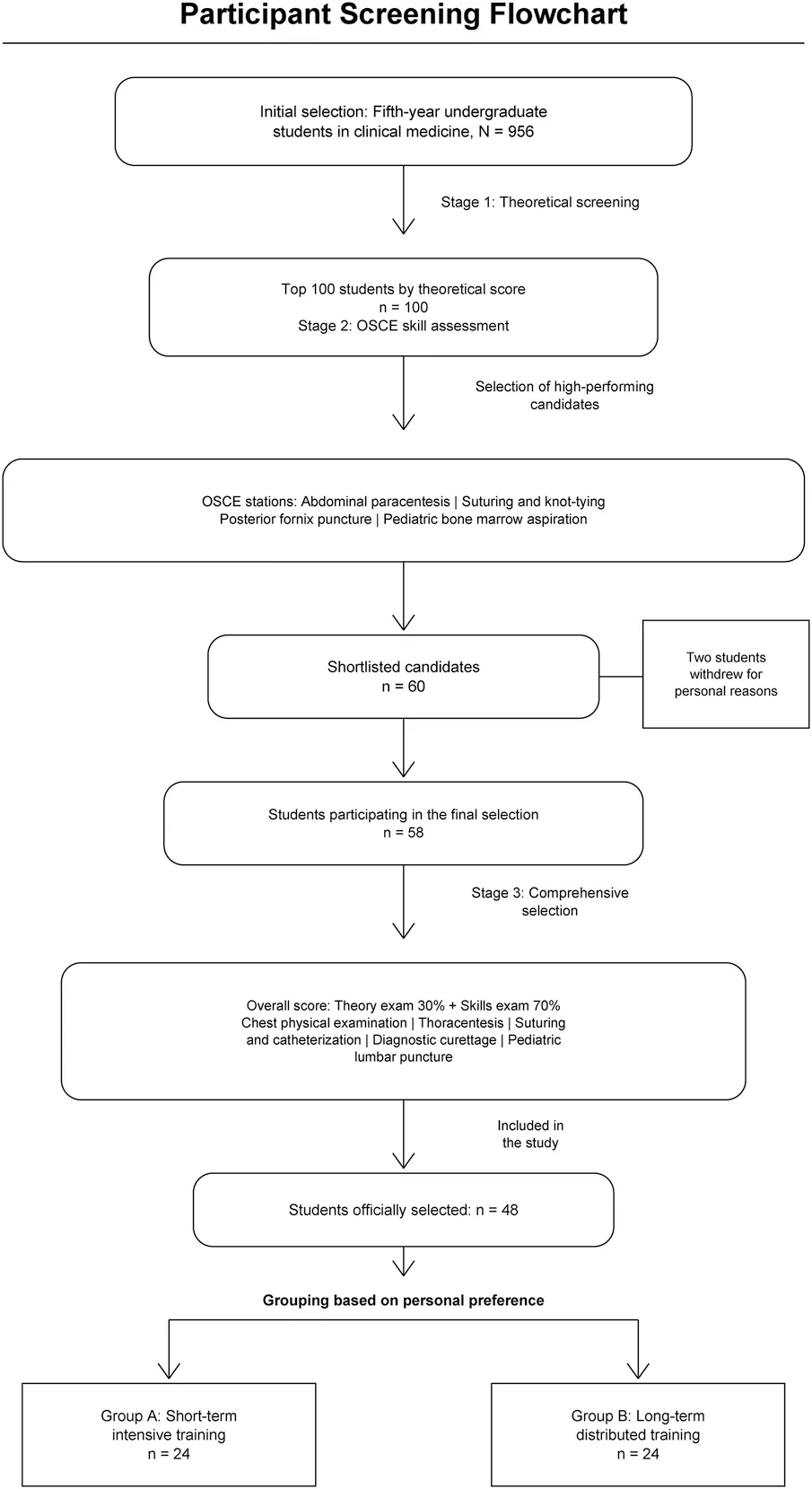
Flowchart of participant screening, enrollment, and group allocation. Participants selected either the 15–day intensive simulation training program (Group A, *n* = 24) or the 5–month distributed simulation training program (Group B, *n* = 24) according to their preferences.

This study adopted a prospective, non-randomized comparative cohort design. Clinical medical interns were prospectively recruited from universities in Shandong Province before January 2025. All eligible participants were fifth-year undergraduate students majoring in clinical medicine with no prior experience of specialized training or participation in similar medical skill competitions. All enrolled students received standardized clinical simulation training starting in January 2025 to prepare for the Shandong Provincial College Student Medical Technology Skills Competition, and the overall participant allocation flow is illustrated in Figure 1.

A total of 48 students were enrolled through voluntary application and institutional selection and were assigned to two groups (*n* = 24 per group) based on their self-selected training schedules. Participants in the intensive training group (Group A) received a 15-day on-site training program with approximately 8 h of daily training, while those in the distributed training group (Group B) underwent a 5-month off-period training program with a maximum of 2 h of daily training. The total training duration was matched between the two groups. All students participated in the competition in teams of three. A total of 28 clinicians from diverse hospital departments were recruited as standardized training instructors.

Primary evaluative outcomes included domain-specific competition scores covering surgery, internal medicine, obstetrics and gynecology, pediatrics, emergency medicine, physician–patient communication, and case analysis. Secondary outcomes consisted of post-competition questionnaire assessments completed independently by students and instructors. Baseline demographic information including age and sex was collected for descriptive analysis. However, several potential confounding variables, such as previous simulation exposure, academic performance, GPA, baseline clinical skill proficiency, and learning motivation, were not systematically assessed at baseline, and thus could not be statistically balanced or adjusted for between-group comparisons. This methodological limitation was considered during result interpretation.

### 2.2 Simulation-based training intervention

All simulation training sessions were conducted in a standardized clinical skills center equipped with high-fidelity training environments, including simulated wards, operating rooms, and emergency units. The training apparatus comprised SimMan 3G high-fidelity mannequins, dedicated task trainers for central venous catheterization and endotracheal intubation, and authentic clinical devices such as defibrillators and mechanical ventilators.

Team-based clinical simulation scenarios were developed strictly in accordance with the official blueprint of the National Undergraduate Clinical Skills Competition. The simulated scenarios covered core clinical competencies, including emergency management (e.g., cardiac arrest, anaphylactic shock), clinical communication (e.g., breaking bad news, clinical handover), and procedural skills (e.g., surgical suturing, lumbar puncture). Each training session was followed immediately by standardized structured debriefing using the advocacy-inquiry framework and plus/delta feedback model to facilitate reflective learning and targeted behavioral correction.

Prior to formal intervention, all 28 instructors from internal medicine, surgery, obstetrics and gynecology, and pediatrics attended a unified standardization workshop to ensure consistent scenario implementation, checklist-based evaluation, and standardized debriefing procedures across all training groups. Group A received intensive training for 8 h per day over 15 consecutive days, with a total training duration of approximately 120 h. Group B received intermittent training for no more than 2 h per day across a 5-month period, with an equivalent total training volume relative to Group A.

### 2.3 Data collection and outcome measurements

Study outcomes were divided into primary and secondary indicators with unified data collection procedures.

Primary outcomes: The overall total score and each domain-specific sub-score obtained from the Shandong Provincial College Student Medical Technology Skills Competition.

Secondary outcomes: A self-developed structured questionnaire was utilized to comprehensively evaluate students’ comprehensive clinical competency after simulation training. The questionnaire items were formulated based on the core assessment dimensions of the National Undergraduate Clinical Skills Competition and the general competency requirements for clinical undergraduates, covering five core domains: specialized knowledge, clinical skills, teamwork ability, emergency response capacity, and integrated professionalism.

All questionnaire items were rated on a 5-point Likert scale (1 = strongly disagree, 2 = disagree, 3 = neutral, 4 = agree, 5 = strongly agree), with higher total scores indicating superior clinical training outcomes. The initial questionnaire was reviewed and revised by experienced clinical teaching experts to eliminate ambiguous items and optimize logical consistency. A preliminary pilot test confirmed excellent internal consistency, with a Cronbach's *α* coefficient greater than 0.8 for the overall scale. All 48 students and 28 instructors completed the questionnaire anonymously and independently, yielding a 100% response rate.

### 2.4 Statistical analysis

All statistical analyses were performed using SPSS 23.0 (IBM, Armonk, NY, USA). Continuous variables were presented as mean ± standard deviation (SD). Independent-samples t-tests were applied for inter-group comparisons of quantitative data. A two-tailed *p*-value < 0.05 was defined as statistically significant.

No prospective sample size calculation was performed, and the sample size of 48 participants (24 participants per group) was determined based on convenient sampling. A *post-hoc* power analysis was conducted based on the actual observed effect size. The results demonstrated a statistical power of 0.75 at an alpha level of 0.05, indicating that the present study was underpowered to detect small effect sizes. This statistical limitation was acknowledged in the final analysis.

### 2.5 Instrument reliability and validity

The self-designed questionnaire applied in this study was developed to systematically evaluate comprehensive clinical competency following simulation training. The item pool was established based on national clinical skill assessment criteria and undergraduate medical education competency standards, ensuring adequate content validity. Each dimension contained refined indicators corresponding to routine training content and competition assessment standards.

To guarantee measurement reliability, expert consultation and item revision were conducted before formal investigation. The finalized scale exhibited good structural consistency and stable measurement performance. The overall Cronbach's *α* coefficient of the questionnaire exceeded 0.8 in the pilot test, indicating satisfactory internal consistency reliability. All participants completed the questionnaire within a unified time frame under anonymous conditions to ensure data authenticity and objectivity.

## 3 Results

### 3.1 General characteristics of students and teachers participating in the survey

Tables 1, 2 summarize the available characteristics of the 48 fifth-year medical students and 28 instructors. Group A completed the 15-day intensive schedule and Group B completed the 5-month distributed schedule before participating in the competition. All 48 enrolled students fully completed the training protocol and subsequent clinical skills competition, yielding a 100% task completion rate. In the formal survey, 48 electronic questionnaires were distributed to student participants and 28 to supervising instructors, with valid response rates of 100% for both cohorts. The groups appeared similar in the reported age and sex distributions; however, these limited descriptors do not establish baseline equivalence because prior simulation exposure, academic performance, GPA, baseline clinical competence, and motivation were not measured systematically. Additionally, each competing student completed a self-assessment of their relevant indicators before and after the training.

**Table 1:** Basic characteristics of students participating in the study.

Characteristic	Group A	Group B
numbers	24	24
Age (years)：Mean ± SD	22.79 ± 1.14	22.8 ± 1.3
Male: *n* (%)	7 (29.2%)	9 (37.5%)

**Table 2:** Basic characteristics of mentors.

Numbers	28
Male:*n* (%)	11 (39.3%)
Age (years): Mean ± SD	40.46 ± 3.44
Intermediate title and below,n (%)	8 (28.6%)
high level professional title,n (%)	20 (71.4%)
Paediatrics, *n* (%)	6 (21.4%)
Internal Medicine Specialties, *n* (%)	10 (35.7%)
Obstetrics and Gynecology, *n* (%)	5 (17.9%)
Surgical Specialties, *n* (%)	7 (25.0%)

### 3.2 Analysis of results from the shandong provincial college students’ medical technology skills competition

**Figure 2:**
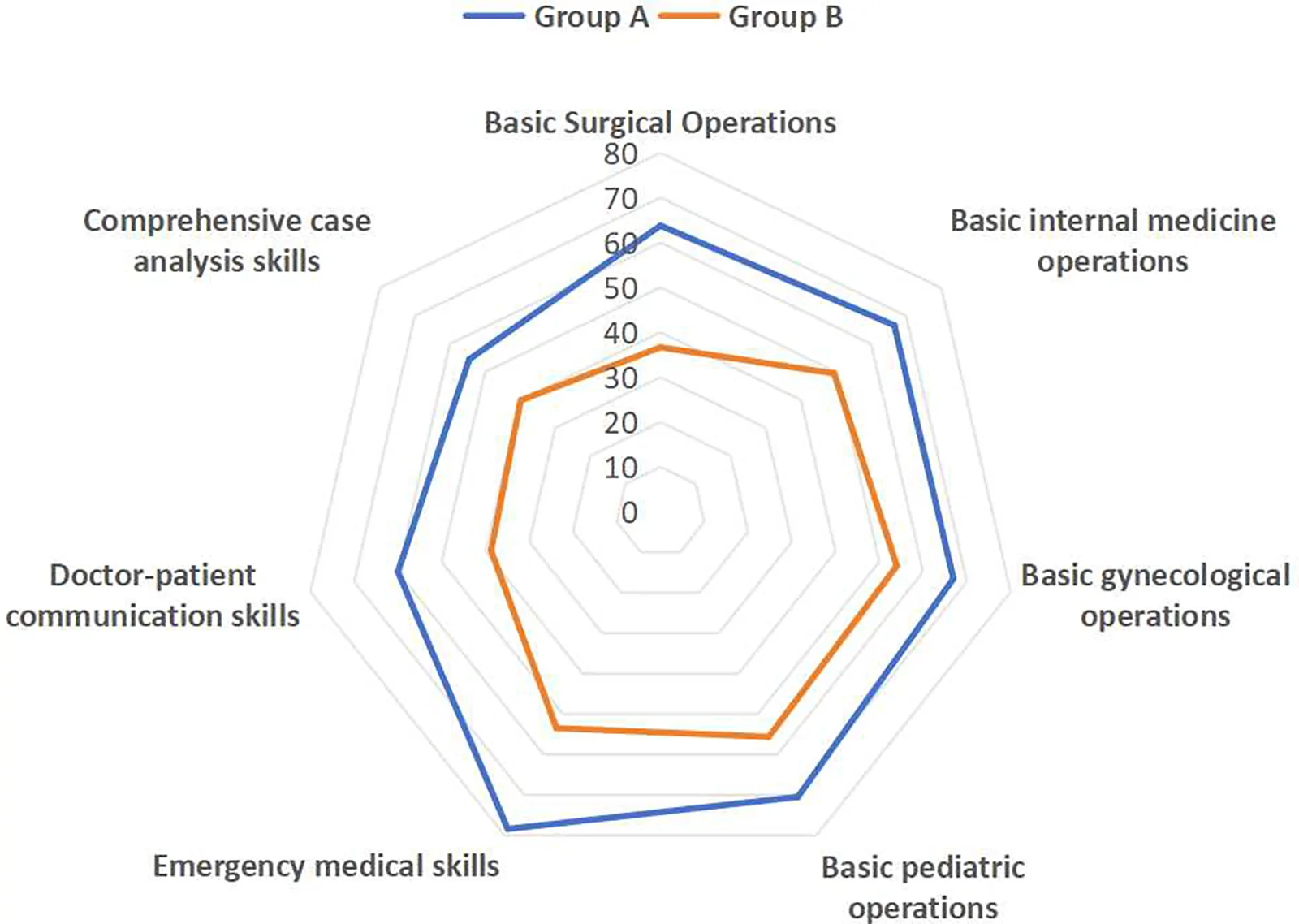
Comparison of clinical skills competition scores between Group A and Group B across the assessed domains.

The competition scores of clinical medicine students in Groups A and B were statistically analyzed using a t-test. The results indicated a statistically significant difference in scores between Groups A and B (*P* *<* *0.05*), as illustrated in Table 3 and Figure 2. This table provides a detailed breakdown of scores across various skill practices, including basic surgical procedures, fundamental internal medicine procedures, essential obstetrics and gynecology procedures, core pediatrics procedures, emergency knowledge and skills, physician-patient communication skills, and case analysis skills.

**Table 3:** Comparison of competition scores between groups A and B.

Competition skill domain	Group A:Mean ± SD (*N* = 8)	Group B:Mean ± SD (*N* = 8)	*t*-value	*P*-value
Basic Surgical Operations	63.7656 ± 13.1265	36.6406 ± 11.2675	2.645	0.019
Basic internal medicine operations	66.6375 ± 14.9493	49.4575 ± 10.6788	4.435	0.001
Basic gynecological operations	66.925 ± 8.12153	54.0062 ± 12.2310	2.489	0.026
Basic pediatric operations	70.50 ± 13.9949	55.6250 ± 6.67485	2.713	0.017
Emergency medical skills	78.4063 ± 12.2539	53.4688 ± 13.2081	3.915	0.002
Doctor-patient communication skills	60.000 ± 13.4104	38.7188 ± 13.1546	3.204	0.006
Comprehensive case analysis skills	54.500 ± 17.7949	39.8438 ± 10.6048	2.001	0.065

### 3.3 Mentor evaluation score analysis

**Figure 3:**
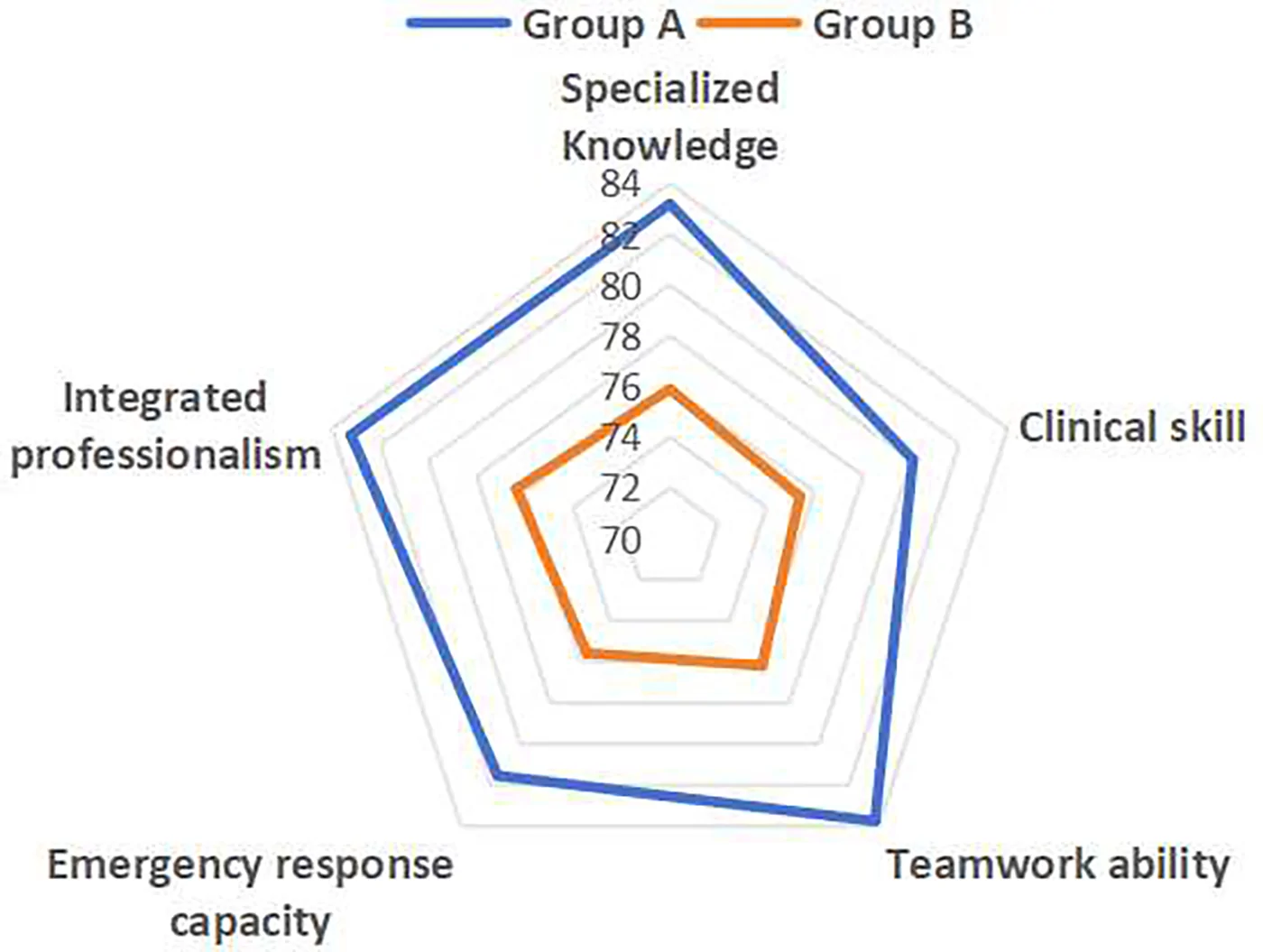
Comparison of instructor evaluation scores between Group A and Group B across specialized knowledge, clinical skills, teamwork ability, emergency response capacity, and integrated professionalism.

Following the competition, we conducted a survey of the instructors guiding the students. A total of 28 instructors from internal medicine, surgery, obstetrics and gynecology, and pediatrics provided comprehensive evaluations of the students’ mastery of professional knowledge, clinical skill proficiency, teamwork capabilities, emergency response abilities, and overall professional competence in Groups A and B. As shown in Table 4 and Figure 3, after undergoing short-term intensive training, Group A students demonstrated significantly greater improvements in clinical skills, teamwork abilities, and emergency response capabilities compared to those who received long-term, short-duration clinical skills training (*p* *<* *0.01*).

**Table 4:** Mentor evaluation score analysis.

Evaluation domain	Group A:Mean ± SD (*N* = 28)	Group B:Mean ± SD (*N* = 28)	*t*-value	*p*-value
Specialized Knowledge	83.18 ± 5.186	75.89 ± 8.999	3.712	0.001
Clinical skill	80.07 ± 4.480	75.39 ± 7.964	2.709	0.010
Teamwork ability	83.75 ± 6.150	76.18 ± 6.313	4.546	0.002
Emergency response capacity	81.54 ± 8.470	75.57 ± 9.122	2.535	0.014
Integrated professionalism	83.21 ± 8.513	76.36 ± 9.318	2.875	0.006
Total points	411.75 ± 15.579	379.39 ± 21.389	6.470	＜0.001

### 3.4 Student self-assessment score analysis

After the competition, all 48 students completed a questionnaire assessing specialized knowledge, clinical skills, teamwork, emergency response, and integrated professionalism. As shown in Table 5, Group A reported higher ratings than Group B across the assessed domains (all *p* *<* *0.05*). Because no validated pre-training measure of these domains was available, these results represent post-training between-group differences rather than demonstrated within-person improvement. Although unquantified baseline differences in self-perceived ability may exist between the two cohorts, the consistent cross-domain advantage observed in post-competition self-evaluations still yields meaningful comparative evidence for evaluating training outcomes within a competition-driven context, and when triangulated alongside objective competition performance metrics and independent faculty assessments, these subjective self-report data collectively provide practical empirical references to guide the optimization of simulation training frameworks for undergraduate medical trainees.

**Table 5:** Student self-assessment score analysis

Self-assessment domain	Group A:Mean±SD (N=24)	Group B:Mean±SD (N=24)	*t*-value	*p*-value
Specialized Knowledge	83.25 ± 5.244	75.83 ± 8.855	3.531	0.001
Clinical skill	79.96 ± 4.759	74.38 ± 8.88	2.715	0.009
Teamwork ability	84.17 ± 5.917	76.46 ± 6.234	4.393	<0.001
Emergency response capacity	81.13 ± 8.694	74.83 ± 9.073	2.453	0.018
Integrated professionalism	83.46 ± 9.079	74.04 ± 9.406	3.529	0.001
Total points	411.96 ± 16.34	375.54 ± 22.432	6.428	<0.001

## 4 Discussion

This study compared immediate, competition-proximal outcomes among medical undergraduates who selected either an intensive or a distributed simulation schedule. Group A had higher competition scores and higher instructor and student ratings than Group B. These findings are associations and do not demonstrate causal superiority of the intensive schedule. Although the groups were similar in the reported age and sex distributions, comparability cannot be assumed: prior simulation exposure, academic performance, GPA, baseline clinical competence, and motivation were unavailable, and preference-based allocation creates substantial scope for residual confounding. Despite these acknowledged methodological limitations, the multidimensional dataset integrating standardized tournament scoring, faculty expert evaluations and student self-perception metrics still delivers actionable empirical insights for clinical skills educators, offering a real-world comparative reference to inform the design and scheduling of undergraduate simulation training within medical schools.

The pattern of results can be interpreted through the three-part theoretical framework introduced above. From a Deliberate Practice perspective, the intensive schedule may have offered repeated, focused practice and rapid instructor feedback close to the competition (9, 10). Experiential Learning provides a complementary interpretation: simulations supplied concrete experience and opportunities for active experimentation, while guided feedback may have supported reflection and conceptual refinement (11). However, the compressed schedule may also have limited reflection and consolidation between sessions, advantages potentially offered by distributed practice. Mastery Learning further suggests that outcomes depend on explicit standards, formative assessment, corrective feedback, and practice until competence is demonstrated—not intensity alone (12). The higher technical, emergency, communication, and teamwork scores in Group A are therefore compatible with feedback-rich rehearsal, but they could also reflect selection, differential engagement, or closer alignment between training and the competition context.

The between-group difference in comprehensive case-analysis scores did not reach the prespecified significance threshold (*p* *=* *0.065*). Advanced case analysis may require accumulated knowledge, reflection, and clinical experience that are less responsive to short, competition-focused rehearsal. This interpretation is tentative, particularly because competition performance was analyzed at the team level with only eight teams per group, limiting precision and statistical power. Instructor ratings were also higher for Group A across specialized knowledge, clinical skills, teamwork, emergency response, and integrated professionalism. Team-based, immersive scenarios plausibly create opportunities for role allocation, information exchange, and coordinated action (6). Nevertheless, instructor ratings are not independent proof of educational effectiveness: instructors were aware of the training format, may have differed in contact time or enthusiasm, and could have been influenced by students’ visible engagement or competition performance. Similarly, post-training professionalism ratings cannot establish that stable professional attitudes, empathy, or ethical judgment changed without validated baseline and follow-up measures.

Notwithstanding these interpretive constraints, the study makes several useful contributions. First, it integrates three complementary sources of evidence: standardized competition scores, evaluations by clinical instructors with relevant expertise, and students’ self-perceived competence. The convergence of these measures across technical skills, teamwork, emergency response, communication, and professionalism provides a multidimensional picture that is more informative than reliance on a single procedural score or confidence measure. Second, the comparison was embedded in an authentic undergraduate training and provincial competition context rather than an experimentally simplified setting. It therefore offers educators practice-based evidence about how two feasible scheduling arrangements perform under real constraints of curriculum time, instructor availability, and assessment preparation. Third, the results identify the outcomes for which intensive scheduling may be most relevant—particularly immediate technical, emergency, communication, and team performance—while the nonsignificant difference in case analysis helps define an important boundary of that potential value. The observed associations between intensive scheduling and higher near-term performance are consistent with deliberate practice theory, which posits that concentrated, feedback-rich rehearsal enhances skill acquisition. The experiential learning cycle—concrete experience, reflection, conceptualization, and active experimentation—was operationalized through high-fidelity scenarios and structured debriefing. Mastery learning principles likely contributed to the intensive group's superior technical performance. Notably, the lack of significant difference in complex case analysis may reflect the need for distributed reflection and consolidation that brief intensive schedules cannot provide. Together, these features provide actionable empirical insight for clinical-skills educators and a real-world comparative reference for designing and scheduling undergraduate simulation training. The consistency across data sources strengthens confidence that the observed pattern is educationally relevant, although it does not eliminate shared-context, expectancy, or selection effects.

Multiple contextual factors and methodological considerations should be interpreted alongside the observed intergroup differences. First, participants self-selected their training track, which may reflect inherent differences in learning motivation, competitive drive, time availability, or informal pre-study preparation across cohorts. While we balanced observable demographic characteristics at baseline, granular pre-intervention metrics such as prior simulation experience, cumulative GPA, baseline clinical proficiency, and self-reported learning motivation were not formally captured to enable statistical adjustment. Second, variations in instructor contact frequency, timely feedback cycles, and faculty engagement naturally emerged between the two training formats; the intensive cohort also received sustained daily supervision, which may have subtly boosted student engagement through heightened awareness of observation (Hawthorne effect). Third, the condensed, high-pressure daily practice routine of Group A closely mirrored the competitive assessment environment, potentially improving participants’ adaptive performance under tournament-style stress. Fourth, interpretability of the results is tempered by a single-center sample, team-level competition scoring, unmasked subjective evaluations, missing standardized pre-training benchmarks, and no long-term follow-up to measure sustained skill retention or real clinical transfer, which modestly limit the precision of effect estimates and broad generalizability.

The practical relevance of the findings lies in informing decisions rather than prescribing a universally superior schedule. When educators face a time-limited objective—such as preparation for a structured skills assessment, an emergency-skills course, or a team-based simulation event—the observed pattern suggests that a concentrated, feedback-rich format is a reasonable option for prioritizing immediate performance. The results also indicate that scheduling decisions should be aligned with the intended outcome: intensive rehearsal may be useful for rapid coordination and procedural execution, whereas complex case analysis, retention, and transfer may require distributed reflection or a hybrid design. Thus, even with residual confounding, the present data can help educators formulate context-specific training plans, allocate instructor time, and select outcomes for local evaluation.

Future studies should build on this real-world comparison by prospectively measuring academic performance, GPA, prior simulation exposure, baseline clinical competence, motivation, and available study time; using randomization where feasible or prespecified adjustment methods when it is not; and documenting contact hours, instructor-to-learner ratios, feedback, content, and mastery standards. Validated baseline, immediate, retention, workplace-transfer, and resource-use outcomes would clarify whether the immediate advantages observed here persist and whether hybrid schedules can combine concentrated rehearsal with distributed reflection and consolidation. Multicenter samples, blinded assessment, video-based behavioral coding, standardized checklists, and qualitative interviews could further distinguish schedule effects from selection, instructor, Hawthorne, and contextual effects.

## 5 Conclusions

In this prospective, non-randomized comparison, participation in a 15-day intensive simulation schedule was associated with higher competition scores and more favorable instructor and student ratings than a 5-month distributed schedule. The findings provide practice-oriented evidence that a concentrated, feedback-rich format may be considered when immediate technical and team performance is prioritized. However, the study's design limitations prevent claims of causal superiority or long-term benefit. Results should inform context-specific educational planning and hypothesis generation for future randomized trials.

## Data Availability

The raw data supporting the conclusions of this article will be made available by the authors, without undue reservation.
