# Supplementary material for: A comparative study of short-term intensive and prolonged low-intensity simulation training on clinical competence among medical students
Source: Front Med (Lausanne). 2026 Sep 1;13:1850843. doi: 10.3389/fmed.2026.1850843 (PMC13574572; doi:10.3389/fmed.2026.1850843)
Supplement: Supplementary file 1 [file Table1.docx]

**Supplementary Materials**

Explanation of Training Measures for Students in Groups A and B

Simulation environment:

The Clinical Skills Training Center of Qilu Hospital, Shandong University, is equipped with advanced and standardized hardware facilities for medical simulation training. It contains dedicated simulated training venues, including simulated operating rooms, ICUs, wards, delivery rooms, and scenario-based emergency training spaces, which can fully reproduce authentic clinical environments. The center is furnished with high-precision training equipment, including high-fidelity whole-organ surgical simulation systems, virtual reality teaching devices, 3D silicone suture modules, and a complete set of multidisciplinary simulation teaching tools. Supported by holographic projection and intelligent assessment systems, the center establishes a full-scenario, intelligent hardware platform for high-standard clinical skill simulation training.

Scenario design:

All participants in this study received standardized clinical simulation training with a unified total training duration of 120 hours. Group assignment differed only in training cycle and intensity, while all training-related variables, including curriculum content, operational standards, and teaching resources, were strictly consistent between groups. The overall training framework was formulated in accordance with the official criteria of the National Undergraduate Clinical Skills Competition, covering core competencies such as standardized physical examination, fundamental clinical operation, emergency resuscitation, clinical reasoning, and team-based collaborative diagnosis and treatment.

Participants were allocated to two distinct training regimens. Group A received short-term intensive simulation training, which consisted of 15 consecutive days of full-time immersive practice with 8 hours of training per day, yielding a total of 120 training hours. Conducted at the standardized Clinical Skills Training Center of Qilu Hospital, Shandong University, this intensive regimen focused on high-frequency competitive clinical skills through standardized operational demonstrations, standardized patient-based scenario simulation, targeted error correction, and repeated skill drills. Daily training rotated through multidisciplinary modules, including internal medicine, surgery, obstetrics and gynecology, pediatrics, and emergency critical care, with staged assessments arranged throughout the training period. In comparison, Group B underwent long-term distributed training across five consecutive months. The training was arranged intermittently in students’ spare time, with a daily training load of less than 2 hours, and the cumulative training duration was maintained at 120 hours, identical to Group A.

The standardized training curriculum for both groups contained four core modules. These modules included multidisciplinary systematic physical examinations across clinical disciplines; basic clinical procedures encompassing surgical asepsis, disinfection and draping, suturing and debridement, venipuncture, urinary catheterization, and multiple diagnostic puncture techniques; emergency and critical care skills, including cardiopulmonary resuscitation (CPR), automated external defibrillator (AED) operation, and emergency airway management; and comprehensive clinical competency training involving clinical case analysis and multi-disciplinary team collaborative rescue practice. All training sessions were supervised and instructed by senior physicians with extensive experience in clinical skill competition guidance to ensure teaching uniformity.

Following the completion of the respective training regimens, all participants underwent identical unified assessments to evaluate clinical competence. The evaluation system combined objective quantitative scores from the official Shandong Provincial Medical Technology Skills Competition and subjective questionnaire assessments. The questionnaires were designed to comprehensively evaluate participants’ professional knowledge mastery, clinical operational proficiency, teamwork competence, and emergency response capabilities.
